# Implementation of a method to visualize noise-induced hearing loss in mass stranded cetaceans

**DOI:** 10.1038/srep41848

**Published:** 2017-02-06

**Authors:** Maria Morell, Andrew Brownlow, Barry McGovern, Stephen A. Raverty, Robert E. Shadwick, Michel André

**Affiliations:** 1 Zoology Department, University of British Columbia, Vancouver, Canada; 2 Laboratori d’Aplicacions Bioacústiques, Universitat Politècnica de Catalunya, BarcelonaTech (UPC), Vilanova i la Geltrú, Spain; 3 Scottish Marine Animal Stranding Scheme, SAC Consulting Veterinary Services, Drummondhill, Inverness, United Kingdom; 4 Namibian Dolphin Project, Walvis Bay, Erongo, Namibia; 5 Animal Health Center, British Columbia Ministry of Agriculture, Abbotsford, Canada

## Abstract

Assessment of the impact of noise over-exposure in stranded cetaceans is challenging, as the lesions that lead to hearing loss occur at the cellular level and inner ear cells are very sensitive to autolysis. Distinguishing ante-mortem pathology from post-mortem change has been a major constraint in diagnosing potential impact. Here, we outline a methodology applicable to the detection of noise-induced hearing loss in stranded cetaceans. Inner ears from two mass strandings of long-finned pilot whales in Scotland were processed for scanning electron microscopy observation. In one case, a juvenile animal, whose ears were fixed within 4 hours of death, revealed that many sensory cells at the apex of the cochlear spiral were missing. In this case, the absence of outer hair cells would be compatible with overexposure to underwater noise, affecting the region which transduces the lowest frequencies of the pilot whales hearing spectrum. Perfusion of cochlea with fixative greatly improved preservation and enabled diagnostic imaging of the organ of Corti, even 30 hours after death. This finding supports adopting a routine protocol to detect the pathological legacy of noise overexposure in mass stranded cetaceans as a key to understanding the complex processes and implications that lie behind such stranding events.

There is an urgent need to develop methods for assessing the effects of underwater man-made noise on cetaceans. High intensity active sonar, and other loud noise sources, for example those from gas exploration, seismic surveys, etc., have the potential to cause lesions to exposed animals. Depending on the distance from the source, injuries can be lethal. As hearing is fundamental to cetaceans, changes to their hearing system may have a large impact on their ability to carry out vital activities. Deriving a forensic protocol to be able to detect if stranded cetaceans have suffered from noise-induced hearing loss is however key to understanding the physiological impact of anthropogenic noise on these species.

Previous studies on terrestrial mammals using electron microscopy demonstrated structural alterations of the organ of Corti as a response to mechanical and metabolic fatigue of the sensory cells, in turn a result of high intensity and long duration sound exposure^1^. Studies on humans showed that similar pathological alterations can be caused by exposure to ototoxic drugs, genetic factors, bacterial and viral infections or age^2^. These alterations include changes in the sensory cell stereocilia and degeneration and loss of the entire hair cell, amongst others^3^,^4^,^5^. When a mammalian cochlear hair cell dies, the neighbouring supporting cells actively participate in the process of hair cell elimination and scar formation. This process, termed “scarring” throughout this paper, comprises the simultaneous expansion and sealing of the reticular lamina^1^,^6^ as a rapid protective response to hair cell apoptosis, and is not to be confused with the more general use of “scar” as a slow healing mechanism involving collagen formation after injury. The presence of scarring among hair cell rows is therefore an important criterion that can be used to assess any possible history of noise-induced hearing loss and these lesions can be differentiated from artefacts that may develop as a consequence of tissue autolysis^7^.

The stereocilia of outer hair cells (OHCs) are in close contact with the tectorial membrane, which is depressed by the tips of the hairs. These depressions in the tectorial membrane are termed imprints. Experimental acoustic overstimulation in guinea pigs resulted in morphological changes in OHC stereocilia imprints on the undersurface of the tectorial membrane^8^. These changes were coincident with a transformation from circular to oval or irregular shape, fusion of adjacent concavities to form a larger concavity and, occasionally, the appearance of filamentous material. Such modifications of stereocilia imprints were found to remain for a minimum of one year in guinea pigs, long after sensory hairs had disappeared.

Quantifying the pathological changes in stranded marine mammals is very challenging due to the sensitivity of the organ of Corti epithelium to autolysis and the numerous potential causes that can lead to similar alterations.

Hearing loss in cetaceans due to age^9^,^10^,^11^,^12^, possible ototoxic drug administration^13^,^14^ or associated with hydrocephaly^15^ have been reported in the literature. However, to the best of our knowledge, evidence of inner ear damage, compatible with noise-induced hearing loss, has not yet been described in a cetacean mass stranding event.

Long-term research has been undertaken to describe the ultrastructure of the normal organ of Corti in toothed whales with electron microscopy^7^. The best described species to date are harbour porpoise and striped dolphin. Describing the morphology of the most apical cells in the organ of Corti (i.e. cells at the tip of the cochlear spiral) is particularly challenging. OHCs in the apex are not firmly supported by Deiters’ cell bodies and the cuticular plate of the OHCs is not enlarged as found in the base or closer to the stapes^7^. This suggests that apical OHCs may be more sensitive to post-mortem decomposition.

Here we present the methodology and findings from analysis of inner ears recovered from two mass stranding events (MSE’s) that occurred in Scotland; in September 2012 and in June 2015. In both cases, to assess potential acoustic causes and auditory pathology, inner ears from the freshest cases were analysed using scanning electron microscopy (SEM).

## Methods and Materials

Individual long-finned pilot whales involved in both MSE’s were necropsied as soon as possible after death. Both ear bones were dissected from the skull, removed and fixed in 10% neutral buffered formalin. Investigation into both MSE’s was coordinated by the Scottish Marine Animal Stranding Scheme (SMASS) with assistance provided from the Cetacean Stranding Investigation Programme, Sea Mammal Research Unit (SMRU) and Moredun Research Institute.

Five teeth were collected from the mid-section of the left mandible of each sampled individual and preserved in 10% normal buffered formalin. Age was estimated for each individual using methods adapted from Lockyer^16^. Results from teeth aging can be found in Table 1^17^.

### 2012 MSE

On September 2^nd^ 2012, 21 long-finned pilot whales (*Globicephala melas*) live stranded and died in shallow rocky foreshore between Anstruther and Pittenweem, East Scotland (56.2147–2.7190). Gross necropsies were carried out on site on all 21 animals over the following 24–48 hours^17^. Six of the freshest samples from a total of 29 ears were extracted and fixed with 10% buffered formalin and considered for analysis (Table 1). Specifically, these were dissected from two juveniles, two sub-adults and two adult individuals. The periotic bone surrounding the cochlea was decalcified using RDO^®^ (Apex Engineering Products Corporation, Aurora, Illinois, USA), a rapid decalcifier based on hydrochloric acid (see decalcification times in Table 1). Concentrations of 50% RDO for the first 24 hours and 25% RDO in the following days were used, according to a previously optimized protocol^18^.

### 2015 MSE

On the 2nd June 2015, 22 long finned pilot whales stranded on Brogaig beach, a steeply shelving, pebble and sand beach on the Trottenish peninsular of Skye (57.6374–6.2255). In total seven animals died or were euthanized by a trained marksman on welfare grounds, and a full necropsy was possible on six cases. One of the animals stranded was observed to be a pregnant female in significant distress and subsequent examination indicated a dystocia with a dead calf in utero. Recovery of carcasses for necropsy was logistically challenging. However, extraction and fixation of the ears from all cases were prioritized. Details for the cases are given in Table 1.

In contrast to the 2012 protocol, the cochlea were perfused with 10% neutral buffered formalin via the round and oval windows prior to the ears being immersed in the fixative. This followed the protocol implemented by Morell and André^19^. Both ears were collected and depending on the extent of post mortem change, one ear per individual or both in case of M161.1/15 that was well preserved, was selected for ultrastructural analysis (Table 1).

The periotic bone surrounding the cochlea was decalcified using 14% ethylenediaminetetraacetic acid (EDTA) tetrasodium salt hydrate at pH 7.4 at room temperature (changing the solution once every 7–9 days^20^; see decalcification times in Table 1).

### Dissection and processing for SEM

The decalcification of the periotic bone was stopped when the vestibular scala and the *stria vascularis* of the cochlea were exposed. Subsequently, the cochleas and their tectorial membranes were dissected, dehydrated with increasing concentrations of ethanol, critical point dried with CO_2_, and then coated with gold-palladium or platinum-palladium. The 2012 samples were observed with an SEM at the Institute of Marine Sciences, Spanish National Research Council, Barcelona, Spain (Hitachi S-3500N). The 2015 samples were observed with an SEM at the University of British Columbia Bioimaging Facility, Canada (Hitachi S-4700).

The same process was followed in all the samples except individual M280.2/12, which was discarded after observing macroscopic advanced autolysis.

To map the findings of our analysis we divided the cochlea into several regions, with the base of the cochlea closest to the stapes and the apex or apical region at the tip of the spiral.

## Results

### 2012 and 2015 necropsy results

A detailed post-mortem investigation was performed to assess likely factors causal or contributory to both MSE’s. Gross and histopathological investigation of all animals did not find any indication of specific disease processes although several animals involved in the 2012 MSE were in poor general health. Toxin burden from harmful algal blooms (HAB) and heavy metal analysis also did not find levels sufficiently elevated to explain the stranding^17^. The observed behavioural response to the apparent dystocia in M161.6/15 was considered a significant factor in the initial stranding of 22 animals in the 2015 MSE.

### Inner ear results: 2012 MSE

**Figure 1:**
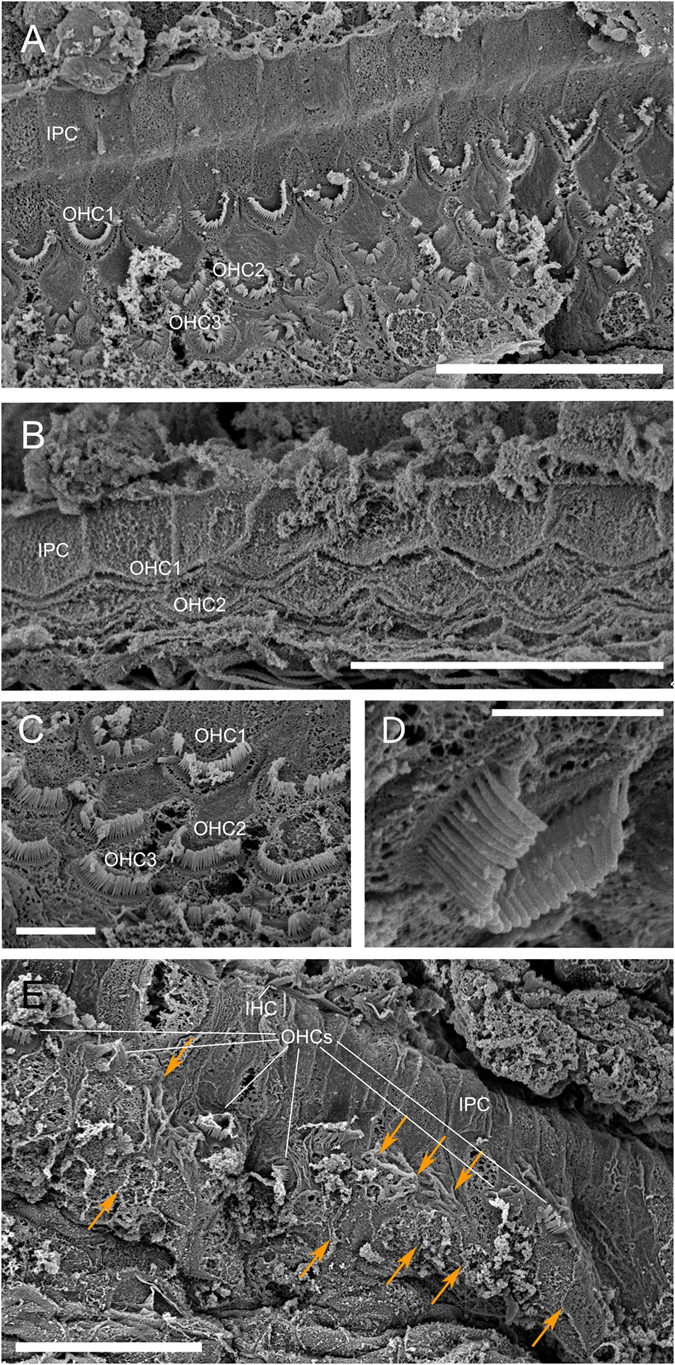
Scanning electron microscope images of the right cochlea from M280.1/12. (**A,C**) Organ of Corti with three rows of outer hair cells (OHCs) in the lower apical turn. (**B**) Organ of Corti in the lower basal turn. (**D**) Detail of an OHC of the upper apical turn. (**E**) Organ of Corti at the apex, with a few OHCs and an area with missing OHCs (highlighted with arrows). IPC: inner pillar cell. Scale bar = 20 μm in (**A,B,E**) 5 μm in (**C**) 3 μm in (**D**).

Based on SEM, we determined that all the cochleas fixed later than 18 hours *post-mortem* (Table 1) were in an advanced state of decomposition that prevented clear observation of the organ of Corti. However, the ear from a case fixed within 4 hours *post-mortem* (M280.1/12) was suitably well preserved; the organ of Corti was present throughout the cochlear spiral (Fig. 1). Although not perfectly intact, OHCs were clearly visible (Fig. 1A–D). However, in the apex of the cochlea, there were missing OHCs in the first 380 μm (arrows in Fig. 1E).

**Figure 2:**
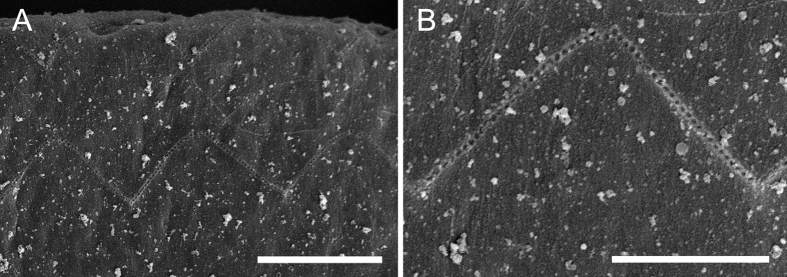
Scanning electron microscopy images of the (**A**) outer hair cell stereocilia imprints on the undersurface of the tectorial membrane of M280.1/12. (**B**) Set of stereocilia imprints from one outer hair cell highlighting its regularity. Scale bar = 5 μm in (**A**); 3 μm in (**B**).

In contrast to the rapid decomposition of the sensory epithelium after death, the tectorial membrane was shown to be more resistant to *post-mortem* autolysis and remained in acceptable condition for analysis when the cochlea was fixed within 18 to 22 hours *post-mortem.* The tectorial membrane showed regular OHC stereocilia imprints on the undersurface along the cochlear spiral (Fig. 2A,B) in all cases analysed, but there was no evidence of imprints in the apical region in any of the individuals sampled.

### Inner ear results: 2015 MSE

**Figure 3:**
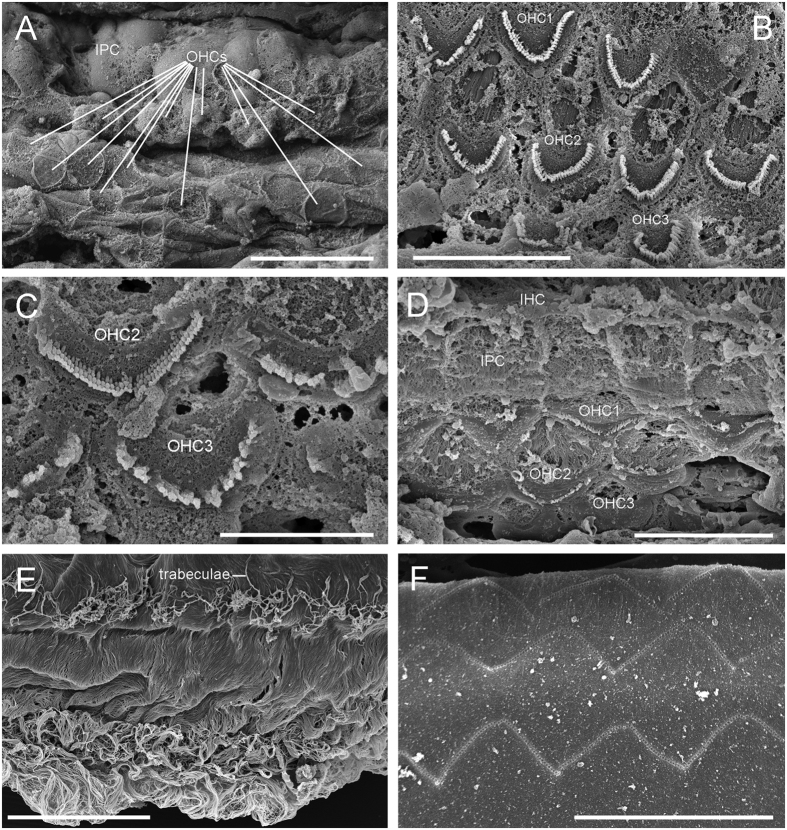
Scanning electron microscope images of four pilot whales cochlea from the 2015 mass stranding in Scotland. (**A**) Apex of the right ear of individual M161.1/15, where the sensory cells had lost their stereocilia but the cellular integrity was apparent. The organ of Corti also had three rows of outer hair cells (OHCs) in the lower apical turn (**B**), upper (**C**) and lower basal turn (**D**). (**E**) Undersurface of the tectorial membrane of the apex showing the Hardesty membrane and highlighting the difficulty of finding the OHC stereocilia imprints. (**F**) Regular imprints of the undersurface of the tectorial membrane. Micrographs (**B,E**) were taken from individual M161.2/15, (**C,F**) from M161.6/15 and (**D**) from M161.3/15 (Table 1). Scale bar = 20 μm (**A**), 10 μm (**B,C and F**), 5 μm (**C**) and 30 μm (**E**). IHC: inner hair cell and IPC: inner pillar cell.

The organ of Corti was found to have varying degrees of decomposition in all the samples (Fig. 3). Not all sensory cells were intact. However, their morphology was clearly visible and there was no conclusive evidence of scarring potentially indicative of noise-induced hearing loss. In general, the ears of the 2015 mass stranding were much better preserved than the 2012 cases, even M161.6/15, whose right ear was perfused 30–36 hours post-mortem (Fig. 3C).

The apical part of the tectorial membrane did not reveal imprints (Fig. 3E), but when apparent in other regions they were regular (Fig. 3F).

## Discussion

Ears extracted from case M280.1/12 showed missing OHCs from the epithelium (arrows in Fig. 1E) in the apex of the cochlea. OHCs are responsible for enhancing auditory sensitivity and frequency selectivity. These missing OHCs would impair the lowest frequency hearing component of the hearing spectrum in M280.1/12. In addition to noise overexposure, the absence of hair cells can be due to several alternative causes, such as age, ototoxic drug administration, polychlorinated biphenyls (PCBs) exposure, congenital or immunological disorders, or species and individual variability in apex morphology^1^,^21^,^22^,^23^,^24^,^25^.

Tooth analysis estimated this animal to be 2.5 years old, hence the absence of OHCs is unlikely to be a function of age. Furthermore, age related hearing loss is observed to primarily affect the high frequencies^21^, encoded at the cochlear base, whereas in this case the OHCs of the base of the cochlea were present with no scarring evident (Fig. 1B). Ototoxic drugs (e.g. gentamicin or amikacin) are similarly known to impair hearing at high frequencies^22^, but it is highly unlikely that the observed cell loss was due to exposure to these compounds, as no medication was administrated to these animals after stranding.

According to studies performed with rats, developmental exposure to PCBs results in severe hearing loss with corresponding mild to moderate loss of OHCs in the upper-middle and apical turns^23^,^24^,^25^. Samples from our 2012 case were analysed for 25 individual PCB congeners using an internationally standardized methodology^26^,^27^. This animal yielded a total concentration of 5.24 mg/kg 25PCBs (on a lipid basis), well below the threshold considered to cause physiological derangements^26^,^28^, thus excluding overexposure to PCBs as a plausible cause for OHC death. Other possible, but highly unlikely, causes such as developmental defects or possible immunological disorders should be considered, however these are not yet detectable in routine necropsy protocols.

**Figure 4:**
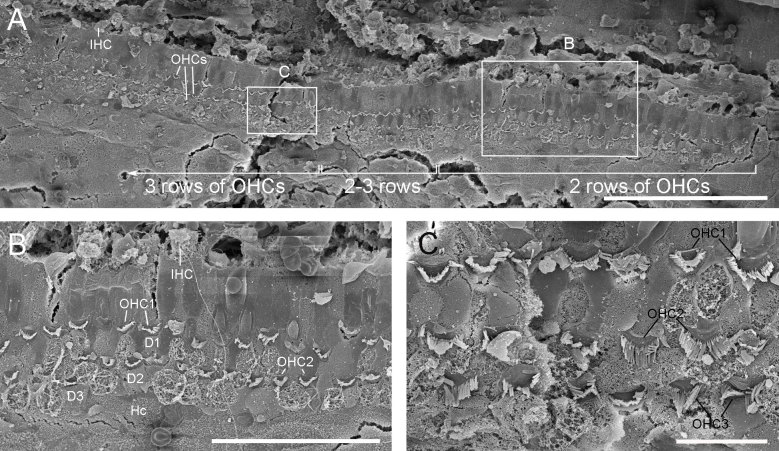
(**A**) Example of the first 500 μm of the apical spiral of the cochlea of a 5 year old non-releasable harbour porpoise from the Vancouver Aquarium (Canada), fixed 10–12 hours post-mortem, using scanning electron microscopy (apex toward the right side). This shows an area with 2 rows of outer hair cells (OHCs), OHC1 and OHC2 in the first 200 μm, then an area of transition with a few missing OHC3 (70 μm), followed by a consistent organ of Corti formed by 3 rows of OHCs. (**B and C**) are higher magnifications of the areas indicated in (**A**). Scale bar = 100 μm (**A**), 40 μm (**B**) and 10 μm (**C**). D: Deiters cells, Hc: Hensen cells.

Very little is known about the common disposition of hair cells in the apex of the pilot whale cochlea. Our analysis of the best preserved pilot whale cochlea from the mass stranding in 2015 (M161.1/15, perfused 15–16 hours post-mortem) did not show this pattern of missing OHCs at the apex (Fig. 3A). The sensory cells had lost their stereocilia at the apex but the cellular integrity was retained and it was possible to differentiate all 3 rows of OHCs. However, studies on terrestrial mammals show a high degree of individual and species variability in apical morphology^29^. More research is needed to establish patterns of morphological normality in pilot whales and other cetacean species. Our preliminary results on harbour porpoises show that some cases there are intermittent missing cells in the third row of OHCs in the first ~200 μm from the apex (Fig. 4) that could be attributed to normal apical variability since the phalangeal processes of the third row of Deiters cells are also missing (Fig. 4B). Although the relatively large area without OHCs observed in the 2012 case M280.1/12, together with the fact that OHCs of the first and second row are affected, is possible evidence of a lesion, individual apical variability in “healthy” animals cannot be ruled out as an alternative explanation.

In the case of noise-induced hearing loss, the sound sources responsible for such scars in individual M280.1/12 would mainly contain low frequency components. Studies of terrestrial mammals showed “the greatest hearing-loss occurs at a frequency about half an octave above the exposure tone”^30^. As the missing OHCs were found in the apex region of the cochlea, the frequency range of the potential sources that could have caused the trauma may correspond to several anthropogenic marine activities, for example, pile driving, geo-physical experiments, or seismic operations. No seismic surveys with sufficient spatio-temporal overlap, that could be considered influential, were reported in the days leading up to the stranding^17^. We have no information on potential sound sources that the individual may have been exposed to at an earlier time.

As expected and previously described in other odontocete species^7^, the tectorial membrane appeared to be more resistant to post-mortem decomposition than the organ of Corti epithelium. The stereocilia of the OHCs fit into depressions or imprints on the tectorial membrane, therefore, any change at the stereocilia level should be reflected in the morphology of the undersurface of the tectorial membrane^8^, although other authors obtained different results when chinchillas were exposed to kanamycin^31^ or noise^32^. The observation that stereocilia imprints throughout the cochlear spiral of all processed individuals were regular (Figs 2 and 3F) except at the apex, would indicate that the OHCs were undamaged before death. This shows there was no evidence of hearing loss in the high frequencies. There were no imprints found in the apex of any tectorial membrane from the 12 samples analyzed. In some cases, its undersurface was not properly exposed when mounted since the tectorial membrane tends to coil during SEM processing, but in other cases, the imprints were not apparent. This result suggests that either 1) pilot whales have a different tectorial membrane apical morphology than terrestrial mammals^33^ where OHC stereocilia might be in close proximity, but not integrated into its undersurface or, more likely, 2) we could not observe the imprints due to the highly fibrous Hardesty membrane of the undersurface of the tectorial membrane (Fig. 3E).

In terms of the 2012 MSE, a scenario where some of the pod was acoustically compromised in their ability to communicate or navigate would offer one explanation for the stranding, but as only animal M280.1/12 was diagnostic along entire cochlear spiral, any causality on the mass stranding cannot be made. Inclusion of accurate inner ear perfusion in the 2015 samples yielded a considerable improvement in preservation of ultrastructural features of the inner ear sensory epithelium. Without perfusion, fixation of pilot whale ears after 18 hours post-mortem did not adequately preserve the organ of Corti cells for ultrastructural evaluation. However, the post-mortem interval of perfused inner ears can extend up to 24–30 hours in pilot whales with adequate retention of ultrastructural detail (Fig. 3A–E). Importantly, this makes the technique applicable to necropsy examinations in a field situation, where logistical constraints often result in a delay between death and ear extraction.

Although many stranding networks are now implementing extraction and fixation of the inner ear in cetacean mass stranding necropsies, it is not yet common practice universally. The results presented here show that an appropriate protocol to recover and analyse ears from suitable candidates is available and may provide essential information to understand the complex processes involved in these acute events. This methodology can be applied to single strandings as well if the time from death to fixation is under 30 hours. Our results reinforce a recommendation presented at the International Whaling Commission workshop on cetacean stranding (San Francisco, December 2015) that carcases are triaged for necropsy, with the tissues most sensitive to post-mortem decomposition, such as the ears, removed first.

Overall, this demonstrates a method whereby ultrastructural changes in the ears of cetaceans can be accurately imaged in wild, dead stranded cetaceans. In turn this allows for quantification of any hearing impairment and, potentially, identification of the frequency range of noise overexposure responsible for ante-mortem hearing loss. This finding is of particular relevance to stranding network responders interested in collecting the ears for potential noise-induced hearing loss diagnosis. In turn this has important implications for policy making and ocean management, especially in those cases when overexposure to anthropogenic sounds may be linked to the cause of a stranding event.

## Conclusions

There is strong demand from the public to establish the reason for cetacean mass strandings. Thorough investigation relies on considering a large number of potential factors with the aim of establishing, when possible, the most probable cause. In many cases the information available may not permit definitive conclusions to be drawn and the significance of a factor or factors has to be interpreted with care so as not to speculate. The inner ear analysis of 2012 and 2015 mass stranding of pilot whales in Scotland revealed that:

1. SEM was particularly useful for detecting lesions or abnormalities in the organ of Corti cuticular plate beyond the first μm from the apex, which can be associated with anthropogenic noise exposure.
2. Perilymphatic perfusion of the cochlea greatly improved preservation of the inner ear ultrastructure, and extended considerably the diagnostic time window in which noise-induced hearing loss could be evaluated in pilot whales.
3. Further research following the methodology presented here on normal apical morphology and the creation of cochlear frequency maps for pilot whales will be essential to distinguish between lesions and normal morphology. In cases of noise-induced hearing loss, these maps will ultimately provide information on the causal sources of such lesions.

## Additional Information

**How to cite this article**: Morell, M. *et al*. Implementation of a method to visualize noise-induced hearing loss in mass stranded cetaceans. *Sci. Rep.* **7**, 41848; doi: 10.1038/srep41848 (2017).

**Publisher's note:** Springer Nature remains neutral with regard to jurisdictional claims in published maps and institutional affiliations.

## Figures and Tables

**Table 1:** Description of the samples analysed.

Id SMASS	year	ear	Time between death and tissue fixation (approx.)	Age group	Age (years)	Total length	Decalcification time
M280.1/12	2012	right	4 hours	sub-adult	2.5	291 cm	2 days 22 h
M280.2/12	2012	left	18–22 hours	adult	26	420 cm	4 days 1 h
M280.4/12	2012	right	18–22 hours	adult	NA	420 cm	4 days 2 h
M280.6/12	2012	left	18–22 hours	juvenile	NA	192 cm	2 days 15 h
M280.7/12	2012	left	18–22 hours	juvenile	NA	191 cm	2 days 22 h
M280.12/12	2012	left	18–22 hours	sub-adult	3	315 cm	2 days 20 h
M161.1/15	2015	right	15–16 hours	adult	42	414 cm	34 days
M161.1/15	2015	left	15–16 hours	adult	42	414 cm	38 days
M161.2/15	2015	right	18–20 hours	adult	9	388 cm	35 days
M161.3/15	2015	left	19.5 hours	adult	24	424 cm	37 days
M161.4/15	2015	left	18–20 hours	sub-adult	5	302 cm	51 days
M161.5/15	2015	left	20–22 hours	juvenile	1	250 cm	35 days
M161.6/15	2015	right	30–36 hours	adult	19	418 cm	35 days

SMASS: Scottish Marine Animal Stranding Scheme^17^.
